# Molecular pathway activation features of pediatric acute myeloid leukemia (AML) and acute lymphoblast leukemia (ALL) cells

**DOI:** 10.18632/aging.101102

**Published:** 2016-11-19

**Authors:** Ivan Petrov, Maria Suntsova, Olga Mutorova, Maxim Sorokin, Andrew Garazha, Elena Ilnitskaya, Pavel Spirin, Sergey Larin, Alex Zhavoronkov, Olga Kovalchuk, Vladimir Prassolov, Alexander Roumiantsev, Anton Buzdin

**Affiliations:** ^1^ D. Rogachev Federal Research Center of Pediatric Hematology, Oncology and Immunology, Moscow, 117198, Russia; ^2^ First Oncology Research and Advisory Center, Moscow, 117997, Russia; ^3^ Moscow Institute of Physics and Technology, Dolgoprudny, Moscow region, 141700, Russia; ^4^ Group for Genomic Regulation of Cell Signaling Systems, Shemyakin-Ovchinnikov Institute of Bioorganic Chemistry, Moscow, 117997, Russia; ^5^ Morozov Pediatric Clinical Hospital, Moscow, 101000, Russia; ^6^ National Research Centre “Kurchatov Institute”, Centre for Convergence of Nano-, Bio-, Information and Cognitive Sciences and Technologies, Moscow, 123182, Russia; ^7^ Pathway Pharmaceuticals, Wan Chai, Hong Kong, Hong Kong SAR; ^8^ Engelhardt Institute of Molecular Biology, Russian Academy of Sciences, Mosow, Russia,119991; ^9^ Department of Biological Sciences, University of Lethbridge, Lethbridge, AB, T1K3M4, Canada

**Keywords:** acute myeloid leukemia, acute lymphoblast leukemia, pediatric, adult, gene expression, intracellular signaling pathways, molecular markers, OncoFinder

## Abstract

Acute lymphoblast leukemia (ALL) is characterized by overproduction of immature white blood cells in the bone marrow. ALL is most common in the childhood and has high (>80%) cure rate. In contrast, acute myeloid leukemia (AML) has far greater mortality rate than the ALL and is most commonly affecting older adults. However, AML is a leading cause of childhood cancer mortality. In this study, we compare gene expression and molecular pathway activation patterns in three normal blood, seven pediatric ALL and seven pediatric AML bone marrow samples. We identified 172/94 and 148/31 characteristic gene expression/pathway activation signatures, clearly distinguishing *pediatric* ALL and AML cells, respectively, from the normal blood. The *pediatric* AML and ALL cells differed by 139/34 gene expression/pathway activation biomarkers. For the *adult* 30 AML and 17 normal blood samples, we found 132/33 gene expression/pathway AML-specific features, of which only 7/2 were common for the adult and pediatric AML and, therefore, age-independent. At the pathway level, we found more differences than similarities between the adult and pediatric forms. These findings suggest that the adult and pediatric AMLs may require different treatment strategies.

## INTRODUCTION

*Acute myeloid leukemia* (AML) and *acute lymphoblastic leukemia* (ALL) are heterogenous diseases of hematopoietic stem cells and progenitor cells. AML is a cancer of the myeloid line of blood cells, characterized by the rapid growth of abnormal white blood cells that accumulate in the bone marrow and interfere with the production of normal blood cells [1]. AML involves high percentages of dedifferentiated and undifferentiated cells, including blasts (myeloblasts, monoblasts and megakaryoblasts) [2]. AML is relatively rare in the childhood, but it is the most common acute leukemia affecting adults, and its incidence increases with age [3]. As an acute leukemia, AML progresses rapidly and is typically fatal within weeks or months if left untreated. AML is cured in 35–40% of people under 60 and in 5–15% of patients over 60 respective-ly [2].

Unlike AML, acute lymphoblastic leukemia (ALL) is most common in childhood, with a peak incidence at 1–6 years of age [4, 5]. AML is characterized by the overproduction and accumulation in bone marrow of immature cancerous white blood cells, referred to as lymphoblasts [6]. Over 80% of the affected children are cured, while only 20-40% of the adults achieve complete remission [4, 7]. Multiple molecular peculiarities, such as diagnostic mutations and certain gene expression signatures, have been associated with AML and ALL in previous studies [8–11].

However, the genetic or gene expression factors responsible for the age-related manifestation features of AML and ALL remain uncertain. To date, gene expression analysis has been performed on a very limited number of AML/ALL cancer samples, especially for the pediatric onset. This may be due to relative rarity of AML/ALL and very limited access to pediatric cancer patient biopsies [12]. On the other hand, it is difficult to compare gene expression data obtained in different experiments and using different experimental platforms, primarily because of well-known batch effect, which reflects experimental bias [13].

In this study, we used microarray hybridization to compare the gene expression in the two groups of human pediatric AML and ALL biosamples. We compared pediatric onset-specific AML gene expression profiles with those characteristic of adult AML. To analyze the expression data, we used molecular pathway approach which was shown to reduce platform-specific bias in various assays [14]. We identified 36 and 172 characteristic pathway and gene expression signatures, respectively, clearly distinguishing ALL, AML and normal cases. We compared the results for pediatric AML with the adult AML and normal blood samples to identify molecular features common and specific for the pediatric and adult disease onset. As the result, we found 7/2 age-independent AML gene expression/molecular pathway signatures and 132/33 those linked with the age-specific AML onset, respectively. These findings shed light on the molecular mechanisms governing age-specific onset of human leukemia and identify novel potential targets for the molecular therapy of ALL and AML.

## RESULTS

### Profiling of gene expression in leukemia samples

The bone marrow biopsy samples were collected and analyzed for seven pediatric acute lymphoblast leukemia (ALL) and seven pediatric acute myeloid leukemia (AML) samples. The mean ages were 7 and 6 years old (y.o.) for the patients in the AML and ALL groups, respectively. Leukemia samples were compared with the normal peripheral blood isolated from the three healthy donors, with the mean age in the group 12 y.o. (Supplementary dataset S1).

The total RNA preps were extracted, and gene expression was profiled with microarray hybridization. We used original customized microchip system developed using the CustomArray (USA) technology of direct electrochemical oligonucleotide synthesis on the array [15]. Using the CustomArray platform, we synthesized the arrays with 2228 oligonucleotide probes corresponding to 2016 human genes involved in 334 intracellular signaling pathways (Supplementary dataset S2). For the custom microchip, we used original oligonucleotide probe sequences of the Illumina HT 12 v4 platform. The library preparation and hybridization protocol is outlined on Supplementary dataset S3. The microarray hybridization signals were quantile normalized according to Bolstad et al. [16], and gene expression data were deposited in GEO database with the accession numbers GSE84574 and GSE84575.

For the adult leukemia samples, we took GEO dataset GSE37307containing 30 AML and 17 peripheral blood samples profiled using the Affymetrix hgu133a microarray hybridization platform. The gene expression data were quantile normalized and further compared with pediatric samples.

### Bioinformatic analysis of gene expression data

We processed the transcriptomic data under investigation to establish normalized cancer-to-normal (CNR) expression rates and to build pathway activation strength (PAS) profiles corresponding to intracellular signaling pathways for each sample. The analysis included 334 pathways (Supplementary dataset S2) and 2016 individual gene products. For PAS measurements, we applied the OncoFinder method which was previously shown to increase the stability of gene expression data [14]. In multiple previous studies, OncoFinder was utilized to analyze human and non-human samples from a broad range of conditions including leukemia, solid cancers, fibrosis, age-related macular degeneration disease, asthma, Hutchinson-Gilford disease and cell culture [17–20]. The formula for PAS calculation for a given pathway (*p)* is as follows: $PAS_{p}=(\sum_{n}ARR_{np}\cdot \text{lg}\left(CNR_{n}\right))/\text{N}$ [21]. The functional role of a gene product in a pathway is reflected here by a discrete flag *activator/repressor role* (*ARR*), which equals 1 for an activator, −1 for a repressor, and shows intermediate values −0,5; 0,5 and 0 for the gene products having intermediate repressor, activator, or unknown roles, respectively. The *CNR_n_* value is the ratio of the expression level of a gene *n* in the sample under investigation to the average expression level in the control sampling. N is the number of individual gene products in the pathway p. The positive value of PAS indicates activation of a pathway, and the negative value - its repression in a biosample under investigation. The CNR and PAS values obtained for the normal and leukemia samples are shown on Supplementary dataset S2.

### Comparison of pediatric and adult normal and leukemia gene expression profiles

The CNR and PAS data were analyzed in two ways to identify gene expression and PAS signatures that distinguish (*i*) pediatric ALL and AML from normal samples and (*ii*) pediatric AML from the adult AML samples.

#### Pediatric ALL-specific features

We compared seven pediatric ALL samples with the three normal peripheral blood samples. To identify the characteristic ALL-specific features, we calculated the “area-under-curve” (AUC) values [22] for the CNR and PAS scores of each of the gene products and pathways under investigation. The AUC value is the universal characteristics of biomarker robustness and it is dependent on the sensitivity and specificity of a biomarker. It correlates positively with the biomarker quality and may vary in an interval from 0.5 till 1. The AUC threshold for discriminating good and bad bio-markers is typically 0.7 or 0.75. The entries having greater AUC score are considered good-quality biomarkers and vice-versa [23]. We could identify 94 gene products and 47 molecular pathways that had close to 1 AUC scores for the ALL-normal comparison (Supplementary dataset S4, Table 1). Among those, branches of Akt signaling [24], cAMP [25], cytoplasmic and mitochondrial apoptosis [26], PTEN [27], ATM checkpoint [28], Hedgehog [29], HGF [30], GSK3 [31], Estrogen and Glucocorticoid reception [32, 33], IGF1R [34], IL2 [35], TNF [36], ILK [37], JAK-STAT [38], JNK [39], mTOR [40], TGF-beta [41], Ras [42], PPAR [43], NGF [44], VEGF [45], Wnt [46], HIF1 and Notch signaling [47] were previously reported in the literature as ALL-associated pathways. However, the identified *GPCR* and *TRAF-associated apoptosis* marker pathways were new, thus representing ~4% of the total ALL-specific pathways.

**Table 1:** Statistics of the gene expression and pathway activation markers identified in this study

Comparison	Gene expression markers (GEM)	AUC (GEM)	Pathway activation markers (PAM)	AUC (PAM)
Pediatric ALL vs Normal	94	~1	47	0.90-1
Pediatric AML vs Normal	148	~1	31	0.95-1
Pediatric AML vs Pediatric ALL	139	0.91-1	34	0.92-1
Pediatric AML vs Pediatric ALL vs Normal	172	0.85-0.98	36	0.84-0.96
Adult AML vs Normal	132	0.75-0.95	33	0.75-0.86

#### Pediatric AML-specific features

When comparing the seven pediatric AML and three normal peripheral blood transcriptomes, we identified 148 marker gene products and 31 molecular pathways with close to 1 AUC scores (Supplementary dataset S5, Table 1). Among them, one top marker pathway (~3%) has not been previously linked with AML: a branch of *Inositol-3-phosphate signaling pathway* responsible for gene expression with the transcriptional factors CREB3, NFATC2 and MEF2D was found to be strongly upregulated in the AML samples in this study. Of note, AML cells also demonstrated several upregulated p53-related apoptosis-promoting pathways: the branch of *Mitochondrial apoptosis pathway* related to the activation of p53-dependent gene expression, the branch of *P53 signaling pathway* responsible for promotion of apoptosis, and the branch of *TNF signaling pathway* responsible for apoptosis. However, this enhanced upstream regulation of apoptosis was blocked by the strongly suppressed downstream branch of *Mitochondrial apoptosis pathway* responsible for the irreversible mechanisms such as the DNA fragmentation (Supplementary dataset S5). A similar figure was seen for the ALL cells, were the activation of the upstream *cytoplasmic* pathway was compensated by the inhibition of the downstream *mitochondrial* apoptosis pathway (Supplementary dataset S4). This phenomenon most likely refers to the overall ability of leukemic cells to block programmed cell death at the downstream stages.

#### AML, ALL and normal peripheral blood-specific features

We next identified CNR and PAS biomarker features that can discriminate between the three classes of pediatric biosamples under investigation: AML, ALL and normal peripheral blood cells, with high AUC scores (Supplementary dataset S6, Table 1). We found 172 such gene products and 36 molecular pathways. Among them, *GPCR*, *CREB* pathways and a branch of *ATM pathway* implicated in cell survival mechanisms, were suppressed in the ALL but upregulated in the AML cells.

#### Comparison of pediatric AML versus ALL samples

**Figure 1:**
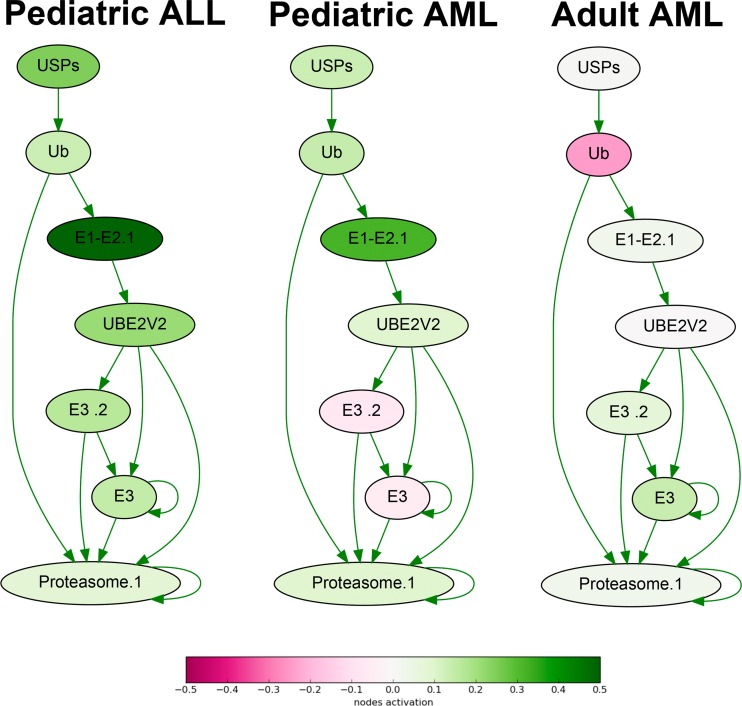
Ubiquitin-dependent proteasome protein degradation pathway shown as an interacting network Pathway activation features are shown for the averaged pediatric ALL, pediatric AML and adult AML transcriptomes. Up-regulated nodes are shown in green, down-regulated - in purple, color legend is provided at the bottom. Saturation of the color is proportional to logarithm of cancer-to-normal (CNR) expression rate for each node of the pathway.

To identify the differential molecular features in the AML and ALL samples, we compared seven ALL versus seven AML gene expression profiles. At the level of gene expression, we found 139 biomarkers with high AUC scores (Supplementary dataset S7, Table 1). Remarkably, this list was enriched by the genes related to proteasome target protein degradation (19/139 genes; Fig.1). All of them were expressed at the high levels in the ALL, and at the significantly lower levels in the AML cells, as this was the case for all eleven *ubiquitin-specific peptidase* (USP) genes from the list, four *ubiquitin protein ligase* genes (*UBR5*, *UBE2T*, *UBE2Q1, UBE2L3*), and four genes for proteasome subunit A (PSMA). There were also five genes for tubulins and associated proteins that were all significantly upregulated in the ALL compared to AML. The same trend was observed for the four *tumor necrosis factor* (TNF) superfamily genes and their receptors (*TNFSF9*, *TNFSF13B*, *TNFRSF10*, *TNFRSF11*).

In contrast, all five genes from the list for *G protein subunits* (*GNA*, *GNB* and *GNG* family genes), five genes for *fibroblast growth factor* (FGF) proteins and three *glutamate metabotropic receptor* (GRM) genes were upregulated in the AML versus ALL samples.

At the molecular pathway level, we identified 34 top features (Supplementary dataset S7, Table 1), including *TNF signaling* pathway, *Ubiquitin-proteasome* pathway (Fig.1), branches of *ATM*, *cAMP*, *Estrogen*, *GPCR*, *HIF-1 alpha*, *ILK*, *IP3*, *MAPK*, *WNT*, and other signaling pathways.

For the first time, our data clearly suggest, that the ALL cells are highly enriched in the proteasomal activities compared to the AML cells. In turn, the AML cells are enriched in GPCR (G protein-coupled receptor) signaling. Those molecular differences clearly seen at both levels of (*i*) individual gene products and (*ii*) molecular pathways, may help to decode the mechanisms for greater curability of the ALL tumors and provide avenues for finding new molecular targets for treating AML in the future. In addition, these data suggest that using of proteasomal inhibitors like Bortezomib may be beneficial also for the treatment of the pediatric patients with the ALL, not only for the AML patients, as this is the case now in several clinical studies [e.g., [48]].

#### Comparison of the adult and pediatric AML-specific features

We next compared 30 and 17 transcriptomes obtained for the adult AML and for the adult normal peripheral blood samples. At the gene expression level, we identified 132 top ranking biomarkers, and 33 – at the pathway level (Supplementary dataset S8, Table 1). These molecular pathways were known to be AML-related and included branches of the *AHR*, *ATM*, *cAMP, FLT3, HGF, ILK, JAK-STAT, Ras, WNT* and other signaling pathways.

In contrast to the pediatric AML samples, where several pathways promoting apoptosis were activated, but blocked at the downstream stages (Supplementary dataset S5), in the adult AML cells the only top pathway related to apoptosis (*mitochondrial apoptosis* pathway) was repressed instead (Supplementary dataset S8). For the top marker genes, we found seven coincidences for both AML types (Table 2), which were all, to our knowledge, not known to be associated with the AML before: *CAMK2B* for Calcium/calmodulin-dependent protein kinase type II beta chain, *EIF4B* for translation initiation factor 4B, *HAPLN1* for hyaluronan and proteoglycan link protein 1, *HIST1H3B* for histone cluster 1, H3b, *LIPE* for hormone sensitive Lipase E, *MAPK13* for mitogen-activated protein kinase 13, and *SAR1B* for secretion-associated Ras-related GTPase 1B. Overall, the top expression biomarkers for the pediatric and adult AML were not highly overlapping, thus producing only two completely matching commonly regulated top pathways: a branch of the *CD40 pathway* influencing cell survival, and a branch of the *Ras pathway* affecting *CDC42 pathway*, which were commonly downregulated and upregulated, respectively, in both types of AML. Both pathways have been previously reported to be associated with the AML [49, 50]. In addition, different branches of the *EGF*, *Glucocorticoid receptor*, *JAK-STAT*, *Mitochondrial apoptosis*, *Ras* and *TGF beta* pathways were also regulated congruently.

**Table 2:** Statistics of the commonly and oppositely regulated gene expression and pathway activation markers in the pediatric and in the adult AML

Matches	Gene expression markers	Pathway activation markers
Complete matches - concordant	(7) *CAMK2B, EIF4B, HAPLN1, HIST1H3B, LIPE, MAPK13, SAR1B*	(2) *CD40 pathway (cell survival)*, *Ras pathway (CDC42 signaling)*
Complete matches - discordant	(4) *BID, F2, JAK3, PPARA*	(1) *ATM Pathway (Cell Cycle Checkpoint Control)*
Incomplete matches – concordant	Not applicable	(14) *EGF Pathway (Cell Survival), EGF Pathway (IP3 Signaling), EGF Pathway (Rab5 Regulation Pathway), Glucocorticoid Receptor Pathway (Cell Cycle Progression)*, *Glucocorticoid Receptor Signaling Pathway (Gene Expression via CREB3, STAT5B, SLC22A2, POU2F1), JAK-STAT Pathway, JAK-STAT Pathway (Nml, SOCS, BCL-XL p21, Myc, Nos2, Gene Expression via STAT2), JAK-STAT Pathway (Gene Expression via MYC), Mitochondrial Apoptosis Pathway (DNA Fragmentation), Mitochondrial Apoptosis Pathway (Apoptosis), Ras Pathway (Receptor Endocytosis), Ras Pathway (Increased T-cell Adhesion), TGF-Beta Pathway, TGF-Beta Pathway (Transciption, Arrested Growth, Apoptosis)*
Incomplete matches - discordant	Not applicable	(17) *ATM Pathway, ATM Pathway (S-phase progression), ATM Pathway (Apoptosis), ATM Pathway (Apoptosis and Senescence), ATM Pathway (Checkpoint Activation), ATM Pathway (G2-Mitosis progression), ILK Pathway (Cell Adhesion), ILK Pathway (Opsonization), ILK Pathway (Cell Cycle, Proliferation), ILK Pathway (Cell Migration, Retraction), Mitochondrial Apoptosis Pathway (Gene Expression via TP53), NGF Pathway (Actin Polymerization, Neurite Outgrowth and Differentiation), NGF Pathway (Neurite Outgrowth and Differentiation), PPAR Pathway, PPAR Pathway (Adipocyte Differentiation, Glucose Homeostasis and Macrophage Function), VEGF Pathway, VEGF Pathway (Actin Reorganization)*.

However, the branches of the *ATM pathway*, *ILK signaling*, *NGF*, *PPAR* and *VEGF* pathways were regulated oppositely in the adult and pediatric leukemia samples (Table 2). These data evidence that the pediatric and adult AML cells differ greatly in gene expression and in molecular mechanisms used to suppress apoptosis and cell cycle arrest, and to promote growth and proliferation.

## DISCUSSION

Acute myeloid leukemia (AML) and acute lymphoblast leukemia (ALL) differ greatly in their behavior, mortality and curability. While ALL occurs primarily in the childhood, the incidence of AML increases with age. The factors that act in an age-dependent manner to promote AML are poorly understood. Although it is widely accepted that the cellular physiology, epigenetic regulation and gene expression of normal hematopoietic stem cells change with age, molecular grounds of such age-dependent cancer transformation remain largely unknown [1]. In the recent review, Karen Keeshan from the University of Glasgow and coauthors said: “Treatment is in general extrapolated from adult AML on the assumption that adult AML and pediatric AML are similar biological entities. However, distinct biological processes and epigenetic modifications in pediatric and adult AML may mean that response to novel therapies in children may differ from that in adults with AML. A better understanding of the key pathways involved in transformation and how these differ between childhood and adult AML is an important step in identifying effective treatment” [1].

In this study, we tried to quantize the distinctions and similarities in pediatric and adult AML at the level of gene expression and molecular pathways. Among the top AML-specific pathways for the groups of adult and pediatric cancers, we identified only three (~9%) completely matching molecular pathways, of which two were commonly regulated (a branch of the *CD40 pathway*, and a branch of the *Ras pathway*), and one (a branch of the *ATM Pathway* governing control over the cell cycle checkpoints) was regulated oppositely, being upregulated in children and downregulated in the adults (Table 2). When considering regulation of the different branches of the same large molecular pathways, we found that there are 14 commonly and 17 oppositely regulated pathways (Table 2). Taken together, these results suggest that the molecular landscapes of the pediatric and the adult AML are very diverse, thus fully confirming the above hypothesis. Our results also open avenue for further in-depth studies decoding functions and roles of the molecular processes identified here in the progression of leukemia and in its age-specific aspects.

In this study, we compared the original experimental and the previously published gene expression data obtained using the custom and the Affymetrix microarray platforms. It is well known that the raw data obtained using different experimental platforms may be poorly compatible with each other [14]. In the future, the adult and the pediatric leukemia samples may be compared using the same experimental platform. However, recently we showed that aggregation of gene expression data into molecular pathways may help to solve the problem of poor data compatibility. For example, deep sequencing and microarray data obtained for the same RNA samples showed generally low correlation (<0.2) when examined at the level of individual genes. However, these correlations improved dramatically, up to 0.9, when activation of molecular pathways was analyzed instead [14]. Here, we compared the experimental and the literature datasets at both the individual gene expression and at the pathway activation levels. We may expect that this granted somewhat greater stability to the results obtained for the comparison of the pediatric and the adult leukemia cells at the level of molecular pathways.

In this study, we identified multiple gene expression and pathway activation markers specific for the AML (Table 1), among them seven genes (*CAMK2B*, *EIF4B*, *HAPLN1*, *HIST1H3B*, *LIPE*, *MAPK13*, *SAR1B*) and one pathway (branch of *Inositol-3-phosphate signaling pathway* responsible for gene expression with the transcriptional factors CREB3, NFATC2 and MEF2D), for which association with the AML was previously unknown.

For the pediatric acute lymphoblast leukemia (ALL), we found 97 gene expression markers and 47 characteristic molecular pathways, of which two (*GPCR* and *TRAF-associated apoptosis* marker pathways) were also new (Table 1). When comparing the pediatric AML and ALL transcriptomes, we identified 139/34 gene expression/pathway biomarkers (Table 1). These results suggest, that the ALL cells are highly enriched in the proteasomal target protein degradation activities compared to the AML cells. In turn, the AML cells are enriched in GPCR (G protein-coupled receptor) signaling. In addition, we found 172 / 36 gene expression/pathway biomarkers that may be used to distinguish between the normal peripheral blood, AML and ALL cells with the high AUC scores (Table 1).

Finally, we generated the high-throughput gene expression profiles for the extremely rare biosamples of human pediatric leukemia biosamples and normal blood, obtained in a single experiment, thus increasing quality of gene expression data. These experiments may contribute to the understanding of molecular grounds that are responsible for the overall phenotypic differences between the pediatric AML and ALL cells, and for their clinical responses.

## MATERIALS AND METHODS

### Tissue specimens and RNA isolation

Three normal peripheral blood specimens from healthy donors, seven AML and seven ALL specimens obtained from patients treated in 2015-16 at the D. Rogachev Center of Pediatric Hematology, Oncology and Immunology (CPHOI), Moscow, Russia, were analyzed. All patients provided written informed consent to participate in this study. This study was approved by the local ethical committee at the CPHOI. The mean age of the ALL patients at the time of sampling was 5.7 years (range 1–14 years), and 7 years for the AML patients (range 1-15 years). The mean age for the healthy donors of peripheral blood at the time of sampling was 11.7 years (range 10–13 years). Both the tumors and normal tissues were evaluated by a pathologist to confirm the diagnosis and estimate the tumor cell numbers. All tumor samples used in this study contained at least 90% tumor cells. Mononuclear cells were extracted shortly after bone marrow or peripheral blood samples collection. Cells were obtained by a density gradient centrifugation method using DiacollTM (Ficoll-1077) (Dia-M, Russia). 2-3 ml of bone marrow or blood was dissolved in PBS up to 10 ml. Cell suspension was layered on 2.5 ml of Diacoll in 15 ml centrifuge tube and centrifuged at 400 RCF for 40 min. Buffy coat was removed and dissolved in PBS up to 14 ml followed by centrifugation at 800 RCF for 10 min. PBS wash procedure was performed twice. Pellet was dissolved in 0,5-1 ml of RNALater solution (Thermo Fisher Scientific). Cells were counted by Scepter™ 2.0 Handheld Automated Cell Counter (Merck Millipore), aliquoted and stored at −20°C till RNA extraction and microarray hybridization. For RNA extraction, cell suspensions with RNALater were centrifuged at 3000 RCF for 5 min. Pellets were dissolved in TRI Reagent (MRC), Direct-zol™ RNA MiniPrep (Zymo Research) was used for RNA extraction. RNA was quantified using Nanodrop (Thermo Fisher Scientific).For the adult leukemia samples, we took GEO dataset GSE37307 containing 30 AML and 17 peripheral blood samples profiled using the Affymetrix HG-U133a microarray hybridization platform.

### Synthesis of microarrays

B3 microarray synthesizer (CustomArray, USA) was used for forty nucleotides-long oligonucleotide probe synthesis on CustomArray ECD 4×2K/12K slides. Synthesis was performed according to the manufacturer's recommendations. Four replicates of total 2228 unique oligonucleotide probes specific to 2016 human gene transcripts were placed on each chip. Chip design was performed using Layout Designer software (CustomArray, USA).

### Library preparation and hybridization

Complete Whole Transcriptome Amplification WTA2 Kit (Sigma) was used for reverse transcription and library amplification. Manufacturers protocol was modified by adding to amplification reaction dNTP mix containing biotinylated dUTP, resulting to final proportion dTTP/biotin-dUTP as 5/1. Microarray hybridization was performed according to the CustomArray ElectraSense™ Hybridization and Detection protocol. Hybridization mix contained 2.5 ug of labeleled DNA library, 6X SSPE, 0.05% Tween-20, 20mM EDTA, 5x Denhardt solution, 100 ng/ul sonicated calf thymus gDNA, 0,05% SDS. Hybridization mix was incubated with chip overnight at 50°C. Hybridization efficiency was detected electro-chemically using CustomArray ElectraSense™ Detection Kit and ElectraSense™ 4×2K/12K Reader.

### Low-level processing of microarray data

Probe signals were geometrically averaged, thus obtaining expression value for each specific type of the probe. The whole dataset was next rounded down to integer values and normalized using the “DeSeq2” package's “estimateSizeFactors” function with respect to the sample type (Normal blood, AML or ALL). The same geometrical averaging was performed for the GSE37307 dataset (excluding the normal testis samples) using the correspondence table from the “hgu133a” Bioconductor package. Then quantile normalization [16] was performed using the “preprocessCore” package, and 2016 genes corresponding to the experimental custom array design were selected for further analysis. Gene expression data were deposited in Gene Expression Omnibus database with the accession numbers GSE84574 and GSE84575.

### Functional annotation of gene expression

The SABiosciences signaling pathways knowledge base (http://www.sabiosciences.com/pathwaycentral.php) was used to determine structures of intracellular pathways, as described previously [51]. We applied OncoFinder original algorithm [21] for functional annotation of the primary expression data and for calculating pathway activation strength (PAS) scores and cancer-to-normal ratios (CNRs). CNR_n_ is the ratio of the expression levels of a gene *n* in the sample under investigation to the average expression in the control group of samples. In this study, we used normalized PAS scores (PAS2), where each initial PAS score obtained according to [21] was divided by the number of genes in the corresponding pathway in order to normalize the activation values and balance heatmap color schemes. Results for the 334 pathways were obtained for each sample. Statistical tests were determined using the R software package.

### Statistical analysis

Hierarchical clustering heatmaps with Euclidean distance and complete-linkage were generated using heatmap.2 function from “gplots” package [52]. Pathways which returned the same PAS scores for all the samples were removed from the analyses. AUC (area under curve) values were calculated using the ‘caTools’ package and cutoff value in each of the comparisons was set to leave approximately 10% of all the gene transcripts/pathways that are the best separators with respect to the given classes. During the triple comparison, three AUC values were calculated for each gene transcript/pathway: Normal vs AML, Normal vs ALL, ALL vs AML, and then averaged to reflect overall separation quality for a given gene transcript/pathway. Average marker PAS/CNR values and the corresponding AUC scores were calculated for each of the sample classes (Normal, AML, ALL) in each comparison.

## SUPPLEMENTARY MATERIAL


















