# Supplementary material for: Molecular pathway activation features of pediatric acute myeloid leukemia (AML) and acute lymphoblast leukemia (ALL) cells
Source: Aging (Albany NY). 2016 Nov 19;8(11):2936–46. doi: 10.18632/aging.101102 (PMC5182073; doi:10.18632/aging.101102)
Supplement: Supplementary file 1 [file aging-08-2936-s001.pdf]

SUPPLEMENTARY MATERIAL

Please browse links in Full Text version of this manuscript to see Supplementary datasets S1-S8.

Supplementary dataset S1. List of the pediatric leukemia patients and normal blood donors investigated in this study.

Supplementary dataset S2. Case-to-normal ratio (CNR) and pathway activation strength (PAS) data calculated for the experimental pediatric leukemia dataset and for the adult leukemia dataset GSE37307.

Supplementary dataset S3. Schematic representation of library preparation and hybridization.
Step 1. Library synthesis. RNA reverse transcription was primed using oligonucleotide primers containing semi-degenerated part at the 3' end and universal sequence at the 5'end. Single strand cDNA was used as a template for complementary strand synthesis using the same oligonucleotide primers. At this step, the library represented overlapping dsDNA fragments flanked by the same universal sequence at both ends.
Step 2. Library amplification and labeling. For library amplification, we used PCR with the universal primers. Labeling of DNA was performed by incorporating biotinylated residuals of dU during amplification. The resulting biotin-labeled dsDNA library was next used for microarray hybridization.

Supplementary dataset S4. Gene products and molecular pathways showing high AUC scores for the pediatric ALL-normal comparison.

Supplementary dataset S5. Gene products and molecular pathways showing high AUC scores for the pediatric AML-normal comparison.

Supplementary dataset S6. CNR and PAS biomarker features that discriminate between the pediatric AML, ALL and normal peripheral blood cells with high AUC scores.

Supplementary dataset S7. Differential CNR and PAS biomarker features in the pediatric AML and ALL samples.

Supplementary dataset S8. Adult AML-specific CNR and PAS biomarker features.
