# Supplementary material for: Molecular pathway activation features of pediatric acute myeloid leukemia (AML) and acute lymphoblast leukemia (ALL) cells
Source: Aging (Albany NY). 2016 Nov 19;8(11):2936–46. doi: 10.18632/aging.101102 (PMC5182073; doi:10.18632/aging.101102)
Supplement: Supplementary file 4 [file aging-08-2936-s004.pdf]

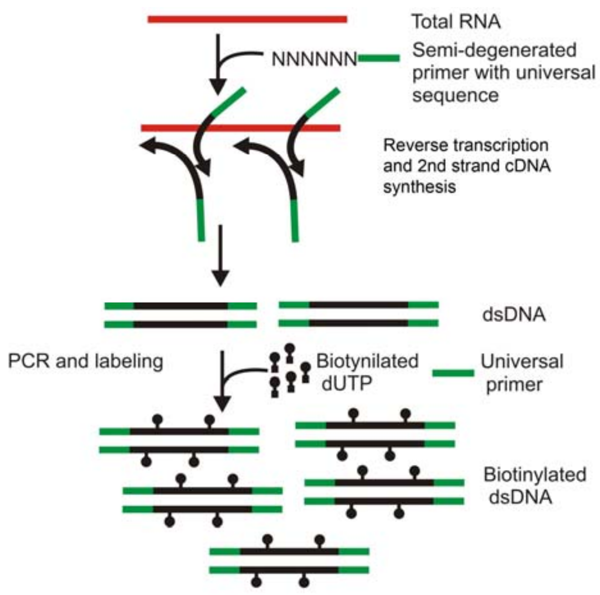

Schematic representation of library preparation and hybridization.

Step 1. Library synthesis. RNA reverse transcription was primed using oligonucleotide primers containing semi-degenerated part at the 3' end and universal sequence at the 5'end. Single strand cDNA was used as a template for complementary strand synthesis using the same oligonucleotide primers. At this step, the library represented overlapping dsDNA fragments flanked by the same universal sequence at both ends.

Step 2. Library amplification and labeling. For library amplification, we used PCR with the universal primers. Labeling of DNA was performed by incorporating biotinylated residuals of dU during amplification. The resulting biotin-labeled dsDNA library was next used for microarray hybridization.
